# O17 What lies beneath: A case of lymphohistiocytic myofasciitis in a 4-year-old girl

**DOI:** 10.1093/rap/rkab067.016

**Published:** 2021-10-19

**Authors:** Dearbhla McKenna, Jenny Curry, Cathy Campbell, Paul Jackson, Madeline Rooney

**Affiliations:** Paediatrc Rheumatology, Musgrave Park Hospital, Belfast, United Kingdom

## Abstract

**Case report - Introduction:**

A 4-year-old girl presented with a 1-year history of difficulty dressing, walking, and getting up from the ground. Examination revealed thickening of the skin, and the presumptive diagnosis was that of scleroderma. However, the dramatic loss of movement seemed out of proportion to her skin changes and therefore further investigations were completed. Her final diagnosis was that of a myofasciitis, a rare diagnosis in a child. This case highlights the importance of a thorough investigative process in order to make the correct diagnosis and explains our management plan for this young girl.

**Case report - Case description:**

A 4-year-old girl, previously well presented with a 1-year history of difficulty dressing, walking, and getting up from the ground. Her joints were never swollen. There were no other systemic symptoms. There was no past medical history. Developmentally all her milestones were achieved on time prior to symptom onset, though she always found it difficult to climb the stairs.

Examination revealed thickening of the skin that felt like advanced scleroderma. The skin on her forearms and ankles was quite turgid. There was an area of morphea around her right shoulder. There were no areas of erythema. There was striking limitation of range of movement of her wrists and shoulders. There was marked stiffness around both ankle joints, although this appeared to be due to the overlying tissue structure, rather than the joint. Diagnosis was likely scleroderma. However, the skin changes seemed out of proportion with the dramatic loss of range of movement.

An MRI of her shoulder girdle revealed dramatic fasciitis involved in the deep fascial planes, just superficial to the muscles. This was marked around the shoulder girdle. Her eosinophil counts gradually climbed. A plausible diagnosis was Eosinophilic Fasciitis/overlap with scleroderma features. This too is unusual in a young child. A muscle biopsy was performed. A full thickness biopsy of her right lateral bicep was taken. This revealed lymphohistiocytic granulomatous inflammation involving the skin, fascia and biceps muscle.

The patient was started on high-dose methylprednisolone, for 3 consecutive days, followed by weaning oral prednisolone and subcutaneous methotrexate.

Two months later her condition was completely transformed. She had gained full range of movement in all her joints. Her skin quality had greatly improved. She remained on methotrexate weekly. A repeat MRI was organised 8 months after commencing treatment. This revealed a dramatic improvement. She remains on methotrexate alone.

**Case report - Discussion:**

Myofasciitis is an unusual inflammatory myopathy almost exclusively described within the adult population. Myofasciitis is a distinctive entity, which is different from the usual idiopathic inflammatory myopathies, sarcoid myopathy and fasciitis-panniculitis syndrome. Clinical presentation can be non-specific particularly with the paediatric population. Hypotonia, developmental delay, fatigue, myalgia and muscle weakness have all been reported. Clinical presentation can be apparent in infancy, or slightly later in childhood.

In macrophage myofasciitis the muscle biopsy typically reveals a monomorphous infiltration of the connective tissue by sheets of densely packed periodic acid-Schiff positive macrophages. There may be intermingled lymphocytes and muscle fibre damage.

A few different associations have been postulated. Intramuscular injections containing aluminium adjunct may be associated with macrophagic myosfasciitis but the underlying process for this remains unclear. The association with autoimmune disease and macrophagic myofasciitis suggests that those from a particular genetic background may be more likely to develop this condition. Genetic predisposition, however, most likely accounts for the variability in the prevalence of macrophagic myofasciitis in different populations.

Given the paucity of literature on macrophage myofasciitis in the paediatric population there is no clear consensus for a treatment strategy. On reviewing the literature many children were first treated with high dose IV pulsed methylprednisolone, followed by oral prednisolone, some received IV Immunoglobulin.

**Case report - Key learning points:**

1. As with many inflammatory muscle disorders patients are often seen by numerous clinicians prior to arriving at paediatric rheumatology. This may mean a prolonged time period before the patient receives appropriate treatment. Through delivering good education to our colleagues mainly in general paediatrics and general practice hopefully this will continue to improve.
2. This case report highlights the importance of a muscle biopsy for diagnosis. Without tissue the correct diagnosis would not be possible and subsequently the treatment given may not be the most suitable for the patient.
3. The histology for this myofasciitis is rare - in adult practice, but definitely within paediatric practice.
4. Methotrexate is well recognised as a suitable and evidence-based treatment for many inflammatory myopathies. Although this condition is slightly different, it has worked incredibly well for the patient. This highlights that when faced with uncertainty as clinicians it is important to consider what we think theoretically will work for our patients.

